# Effects of Abortion Legalization in Nepal, 2001–2010

**DOI:** 10.1371/journal.pone.0064775

**Published:** 2013-05-31

**Authors:** Jillian T. Henderson, Mahesh Puri, Maya Blum, Cynthia C. Harper, Ashma Rana, Geeta Gurung, Neelam Pradhan, Kiran Regmi, Kasturi Malla, Sudha Sharma, Daniel Grossman, Lata Bajracharya, Indira Satyal, Shridhar Acharya, Prabhat Lamichhane, Philip D. Darney

**Affiliations:** 1 Department of Obstetrics, Gynecology and Reproductive Sciences, Bixby Center for Global Reproductive Health, University of California San Francisco, San Francisco, California, United States of America; 2 Center for Research on Environment Health and Population Activities (CREHPA), Kathmandu, Nepal; 3 Department of Gynecology and Obstetrics, Tribhuvan University Teaching Hospital, Institute of Medicine, Kathmandu, Nepal; 4 Bharatpur District Hospital, Chitwan, Nepal; 5 Department of Gynecology and Obstetrics, Medicare National Hospital and Research Centre, Chabahil, Kathmandu, Nepal; 6 Former Secretary, Ministry of Health and Population, Kathmandu, Nepal; 7 Ibis Reproductive Health and University of California San Francisco, San Francisco, California, United States of America; 8 Former Director Paropakar Maternity and Women’s Hospital, Current Director and Chair Capital Hospital, Kathmandu, Nepal; 9 Paropakar Maternity and Women’s Hospital, Kathmandu, Nepal; 10 Lumbini Zonal Hospital, Butwal, Nepal; Institut Jacques Monod - UMR 7592 CNRS - Université Paris Diderot, France

## Abstract

**Background:**

Abortion was legalized in Nepal in 2002, following advocacy efforts highlighting high maternal mortality from unsafe abortion. We sought to assess whether legalization led to reductions in the most serious maternal health consequences of unsafe abortion.

**Methods:**

We conducted retrospective medical chart review of all gynecological cases presenting at four large public referral hospitals in Nepal. For the years 2001–2010, all cases of spontaneous and induced abortion complications were identified, abstracted, and coded to classify cases of serious infection, injury, and systemic complications. We used segmented Poisson and ordinary logistic regression to test for trend and risks of serious complications for three time periods: before implementation (2001–2003), early implementation (2004–2006), and later implementation (2007–2010).

**Results:**

23,493 cases of abortion complications were identified. A significant downward trend in the proportion of serious infection, injury, and systemic complications was observed for the later implementation period, along with a decline in the risk of serious complications (OR 0.7, 95% CI 0.64, 0.85). Reductions in sepsis occurred sooner, during early implementation (OR 0.6, 95% CI 0.47, 0.75).

**Conclusion:**

Over the study period, health care use and the population of reproductive aged women increased. Total fertility also declined by nearly half, despite relatively low contraceptive prevalence. Greater numbers of women likely obtained abortions and sought hospital care for complications following legalization, yet we observed a significant decline in the rate of serious abortion morbidity. The liberalization of abortion policy in Nepal has benefited women’s health, and likely contributes to falling maternal mortality in the country. The steepest decline was observed after expansion of the safe abortion program to include midlevel providers, second trimester training, and medication abortion, highlighting the importance of concerted efforts to improve access. Other countries contemplating changes to abortion policy can draw on the evidence and implementation strategies observed in Nepal.

## Introduction

An estimated 5 million women are hospitalized for abortion-related complications per year in the developing world [1]. Rates of unsafe abortion are rising worldwide, and the legal status of abortion is associated with the risk of maternal morbidity and mortality [2]. While policy conditions on abortion have become more restrictive in many countries, some have recognized the public health potential of a more liberal policy. In Nepal, legislation making abortion legal was passed in 2002, supported by advocacy efforts highlighting very high maternal mortality in the country, much attributed to unsafe abortion [3]. Previously, induced abortion in Nepal was equated with homicide, and punished with imprisonment [4]. The law now permits women to request abortion up to 12 weeks gestation, up to 18 weeks for rape or incest, and with a physician’s approval at any stage of pregnancy to protect mental or physical health and in cases of fetal anomaly [5]. Sex-selective abortion is prohibited, and adult consent is required for girls less than 16 years old.

In 2004, the first certified abortion clinic opened, followed by steady expansion of services. The Nepali government, in partnership with non-governmental organizations, instituted a nationwide program to train abortion providers and regulate the safety and availability of care [6]. Initially, training in manual vacuum aspiration (MVA) was offered only to physicians; however, starting in 2008, staff nurses and auxiliary nurse midwives became eligible to perform MVA up to 8 weeks. Second trimester abortion training and certification for physicians began in 2007, and in 2009 medication abortion was added to the safe abortion program. By the end of 2011, over 1,200 clinicians and 532 clinical sites were trained and certified [6]. Many women are still not aware of the change in the legal status of abortion, or have erroneous beliefs about the law (e.g., husband’s permission needed) [7], but the change in legal status and highly organized implementation efforts allowed nearly 500,000 women to obtain safe, legal abortion care by 2011 [6]. Maternal mortality is estimated to have declined from 360 to 170 per 100,000 live births from 2000 to 2010, although limitations in the available data for estimating maternal mortality have been noted [8], [9]. Other approaches for assessing the role of abortion legalization in women’s health are needed, and despite certain design limitations, hospitals treating post-abortion care cases can provide important insight.

We assess serious abortion complications over a ten year period spanning the legalization of abortion (2001–2010). The study is modeled on those conducted in the United States and elsewhere, where declines in mortality and morbidity from abortion were observed after legalization [10]–[13]. Studies in gynecology/obstetrics wards, particularly in hospitals with large service areas, yield valuable information on unsafe abortions practices, and tend to capture the most severe abortion complications occurring in a population [14]–[16]. We hypothesized that a decline in serious abortion complications would occur at major referral hospitals in Nepal, following implementation of the abortion law.

## Methods

### Ethics Statement

The study protocol was approved by the Committee on Human Research at the University of California, San Francisco and the Nepal Health Research Council. The study relied on retrospective review of medical charts, and did not include abstraction of personal identifiers, therefore, the institutional review boards waived the need for written informed consent from the patients, for their information to be stored in the hospital database and used for research.

### Study Design

This study is a retrospective medical chart review of all abortion-related admissions occurring from January 2001 through December 2010 at four large, public referral hospitals, serving predominantly poor women most likely to experience unsafe abortion. The largest public maternity hospital (415 beds), Paropakar Maternity and Women’s Hospital (MH), is located in the Kathmandu metropolitan area, where nearly 10% of the population of Nepal resides. It is a central referral hospital for maternal and neonatal care, receiving patients from the entire country. Tribhuvan University Teaching Hospital (TUTH) in Kathmandu is the largest and oldest academic public hospital in Nepal (444 beds), and receives referrals for serious and complicated gynecological admissions from all regions of the country. Two public referral hospitals outside of the Kathmandu Valley are also included: Lumbini Zonal Hospital (LZN) (150 beds) and Bharatpur District Hospital (BDH) (430 beds). Located in the populous Terai region, and serving post-abortion care patients from urban, peri-urban, and rural areas, they are important regional referral centers for populations in adjoining districts: LZN sees cases from 11 districts containing 14% of Nepal’s population and BDH sees cases from 8 districts with 14% of the population.

### Medical Chart Review Protocol

To identify abortion complication cases, we reviewed all gynecological admissions as well as maternal and neonatal mortality cases presenting to all units of the hospitals, including emergency, post-abortion care, gynecology and obstetrics. A detailed flow chart was used to identify cases of spontaneous and induced abortion (≤28 weeks gestation) and abortion-related complications. We used broad criteria to ensure capturing cases that may have been poorly documented before legalization. To determine eligibility, diagnostic fields were reviewed and charts indicating abortion were abstracted (threatened, inevitable, incomplete, complete, and septic; spontaneous and induced). When a diagnostic field did not specify abortion, but was suggestive of abortion, other fields were reviewed to assess eligibility. Likely abortion complications, such as repair of uterine perforation, were abstracted and further evaluated for eligibility before analysis. In cases of questionable eligibility, a senior obstetrician-gynecologist or senior nurse involved in post-abortion care at the hospital reviewed the chart [17].

Eligible charts were abstracted using a study form containing fields for demographic characteristics, reproductive history, contraceptive use, clinical assessment on admission and during hospitalization, treatments received, and outcome. Charts for patients receiving elective abortion services at the hospital were not screened unless the patient was later hospitalized.

Trained research assistants with medical backgrounds conducted the chart review from 2007 to 2011. A pilot study of 1,014 charts from the years 2001–2005 (at TUTH and MH) was conducted to test and refine the eligibility determination process and study instruments. For example, during the pilot study we verified that obstetric ward charts did not contain abortion cases, and focused our data collection on gynecology ward charts. We also eliminated and added fields and response categories to the abstraction tool based on data availability. Throughout data collection, random samples of registry entries and abstraction forms were compared to the original medical charts to ensure accuracy. Abstraction forms were manually coded and data were entered and checked for consistency in the database program dBase IV (Binghamton, NY). Inconsistencies were compared with the original medical chart and corrected. Cleaned data were transferred into Stata v11.1 (College Station, TX) for analysis.

Before analysis, cases that did not reference abortion in the chart were evaluated to assess whether they were likely abortion-related complications. For example, the protocol required abstraction of all cases of uterine repair, but some were for vaginal prolapse. Along with cases determined not to be abortion complications (n = 580), hydatidiform mole (n = 410) and ectopic pregnancy (n = 192) were excluded. At TUTH, data are from April 2002 through December 2010 because the hospital disposed of records after 5 years.

### Definition of Serious Abortion Complications

The study outcome is the proportion of serious complications, relative to all abortion complication cases. Our analysis includes complications arising from spontaneous and induced abortion, because they cannot be accurately differentiated [18]. Women are often reluctant to disclose attempted induced abortion and it is not always documented in medical charts, especially in an illegal context [19]. Health complications from spontaneous abortion are likely to remain relatively constant in the population, whereas those arising from induced abortion should decline with safer care since sepsis and injuries are primarily due to unsafe induced abortion procedures. We assessed the proportion of serious abortion complications relative to all abortion complications presenting to help account for secular trends in fertility, health care use, and abortion, and to test whether a shift toward less serious health complications would occur following abortion legalization. To check our results, we also analyzed the outcome of proportion of serious abortion complication relative to live births over the time period.

We adapted a categorization scheme proposed by others to code the severity of complications using clinical signs and symptoms to distinguish between uncomplicated incomplete abortion cases and those with more serious health implications [20]. Our approach differed only slightly: we did not code cases with any sign of interference as high severity, and we did not attempt to distinguish between low and medium severity cases. Higher severity cases of infection, injury, or systemic complications have recorded temperature of 102°F or above, a pulse of 120 beats per minute or more, septic shock or septicemia, hypovolemic shock, generalized and local peritonitis, endometritis, pelvic or genital tract infection, a retained foreign body or injury from abortion, organ failure, loss of consciousness, or death. A separate variable was constructed to identify cases where induced abortion was explicitly documented in the medical chart.

### Analysis

Complications during three periods were described with counts and frequencies: before implementation (2001–2003), early implementation (2004–2006), and later implementation (2007–2010). Types of complications were compared across the three time periods with the Fisher Exact test for categorical differences. Tests for trend for each phase were conducted using segmented Poisson regression with flexible splines fit to the time periods [21], [22]. Count of cases per month is the dependent variable with time as an independent variable representing the incidence-rate ratio (IRR) with the natural log of total cases as an offset variable. We used splines to test the trend in slope for each time period. Marginal splines were used to compare changes in the rate of increase or decrease from the previous period. Multiple variable logistic regression models were also used to estimate the odds of a serious abortion complication by time period, adjusted for and testing the odds of risk by stage of pregnancy at admission, patient age, and whether induced abortion was documented in the medical chart. We also assessed the odds of sepsis, the most common complication of unsafe abortion, by these factors. The multivariable logistic regression models were also adjusted for season (spring, summer, winter, fall) and hospital. Logistic models estimated only on the documented induced abortion cases were also tested to check if results were consistent with the main findings.

## Results

Of 24,676 cases abstracted, 23,493 cases of abortion complications were included in the final analytic sample; 16,499 cases were from MH, 3,135 cases were from LZN, 2,952 were from BDH, and 907 cases were from TUTH. The mean age of the women presenting with abortion complications was 25.4 (SD = 6.1), and nearly all were married (Table 1). Nearly one-third of women were nulliparous, and over one-third had had two or more births. Induced abortion was documented in the medical chart in 9.6% of cases overall, and higher (11.5%) during later implementation.

**Table 1:** Characteristics of patients abstracted from medical charts for all abortion complication cases, 2001–2010, N = 23,493.

Characteristic	Total^a^
Mean age (SD)	25.4 (6.1)
n = 23,471	
Unmarried (%)	0.6
n = 22,247	
Mean years married (SD)	5.7 (6.0)
n = 16,046	
Religion (%):	
n = 18,466	
Hindu	96.5
Non-Hindu	3.5
tNumber of births (%):	
n = 17,744^b^	
None	32.2
One	32.0
Two or more	35.8
Gestation on admission (%):	
n = 21,713	
4–2 weeks	73.9
13–18 weeks	16.1
19–28 weeks	10.0
Induced abortion (%)^c^	9.6
n = 23,493	

a Information on patient demographics was not consistently available in the medical chart. The percentages are calculated on the non-missing n.

b Missing data on the number of births increased at MH and TUTH, and declined at BDH and LZH.

c Clinical evidence such as foreign body or injury from instrumentation (e.g., uterine perforation) or free-text chart documentation of induced abortion based upon patient disclosure or provider observation.

From 2001 to 2010 the number of abortion complications presenting at hospitals rose, ranging from a low of 2,120 in 2002 to a high of 2,948 in 2010. This is consistent with a secular increase in health care use occurring over the time period, particularly in the most recent years when no-cost services at government clinics were instituted [23]. A review of total hospital admissions, live births, and gynecological admissions at the hospitals also reflects this increase in health care use. At MH, for example, in 2001 there were 21,957 admissions and in 2010 there were 29,312 admissions. The mean proportion of gynecological cases that were abortion-related remained stable over time (0.45, 95% CI 0.44, 0.46).

**Figure 1:**
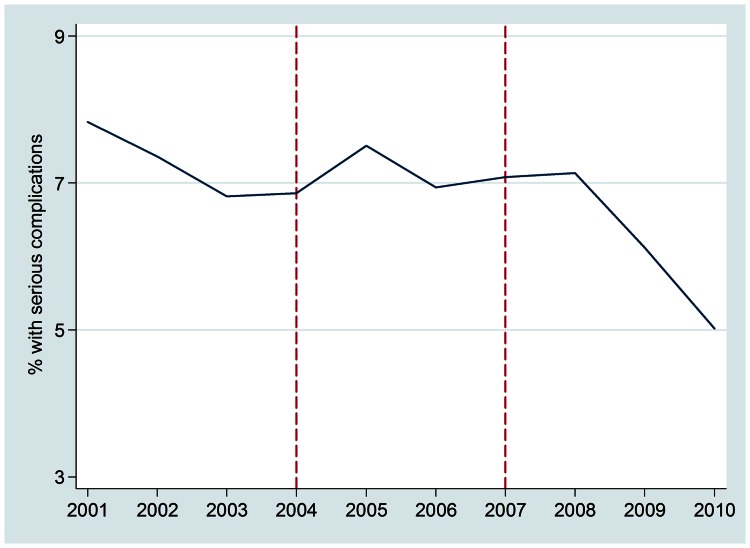
Trend in the percentage of abortion cases with serious complications presenting at four tertiary care hospitals in Nepal, 2001–2010, N = 23,493. Early implementation, monthly IRR from segmented Poisson regression = 1.002, p<.001; later implementation, monthly IRR = 0.993, p<.001; marginal spline test for slope change, p<.001) Serious complications are those with infection (sepsis or septic shock, peritonitis, endometritis, severe pelvic or peritoneal infection, or body temperature> = 102 F), evidence of foreign body or mechanical injury, systemic complications, such as organ failure, or death. 2002 - Passage of legal abortion legislation 2004 - First trimester services and trainings begin 2007 - Second trimester services and trainings begin 2008 - Midlevel providers trained and legally provide first trimester abortion 2009 - Medication abortion added to safe abortion program.

The overall proportion of serious abortion complications relative to all complications was lowest in the later implementation period. Figure 1 illustrates the emergence of a decline in 2007 that steepens after 2008. An increasing trend in all serious complications was observed during early implementation, whereas a significant decrease occurs in the later period (p<.001). There was a significant overall decline in the proportion of total complications and septic abortion cases across the ten year study. Declines were greatest for sepsis and for systemic complications (Table 2).

**Table 2:** Serious abortion complications as a proportion of all abortion complications presenting at hospitals before and after implementation of legal abortion in Nepal, N = 23,493.

	*Before implementation* *2001–2003 N = 6,486 % (n)*	*Early implementation* *2004–2006 N = 6,863 % (n)*	*Later implementation* *2007–2010 N = 10,144 % (n)*
**Any serious complication, total** *	**7.34 (476)**	**7.11 (488)**	**6.25 (634)**
**Infection** *	**5.06 (328)**	**4.23 (290)**	**4.22 (428)**
Sepsis***	3.96 (257)	2.49 (171)	2.11 (214)
High fever (≥102°F)***	1.50 (97)	2.13 (146)	2.44 (248)
Pelvic infection	0.28 (18)	0.25 (17)	0.32 (32)
Peritonitis	0.19 (12)	0.29 (20)	0.21 (21)
Endometritis	0.00 (0)	0.03 (2)	0.02 (2)
Other infection^a^	0.06 (4)	0.04 (3)	0.07 (7)
Injury**	**0.22 (14)**	**0.60 (41)**	**0.44 (45)**
Uterine***	0.12 (8)	0.50 (34)	0.36 (37)
Vaginal/perineal	0.06 (4)	0.09 (6)	0.03 (3)
Intestinal	0.02 (1)	0.10 (7)	0.05 (5)
Cervical	0.02 (1)	0.01 (1)	0.04 (4)
Foreign body	0.03 (2)	0.09 (6)	0.06 (6)
Systemic***	**2.87 (186)**	**3.50 (240)**	**2.36 (239)**
High pulse (≥120 bpm)^b^	1.60 (104)	1.81 (124)	1.43 (145)
Shock* c	0.43 (28)	0.61 (44)	0.27 (34)
Acute renal failure	0.06 (4)	0.04 (3)	0.06 (6)
Multi-organ damage/failure	0.05 (3)	0.06 (4)	0.02 (2)
Disseminated intravascular coagulation (DIC)	0.05 (3)	0.00 (0)	0.01 (1)
Acute respiratory distress syndrome (ARDS)	0.02 (1)	0.00 (0)	0.01 (1)
Cardiac failure	0.02 (1)	0.00 (0)	0.01 (1)
Other systemic^d^	0.17 (11)	0.25 (17)	0.16 (16)
**Death**	**0.06 (4)**	**0.06 (4)**	**0.04 (4)**

* p<0.05, **p<0.001, ***p<0.001, from Fisher’s Exact statistical test for categorical differences.

a Other infections include peritoneal infection and pyoperitoneum, as well as two cases of unspecified infection treated with intravenous antibiotics.

b Includes high pulse related to systemic complications, blood loss, and unspecified.

c Not including septic shock.

d Other systemic complications include deep vein thrombosis, embolism and loss of consciousness.

Multiple variable analyses of the risk of serious complications for each time period show a significant reduction in risk for the later phase of implementation compared to the period before implementation of legal abortion (OR = 0.74, p<.001) (Table 3). An even greater reduction in the odds of serious complications was observed among documented cases of induced abortion (OR = 0.49, p<.001). Risk of sepsis was lowest during later implementation (OR = 0.37, p<.001), but was also significantly lower during early implementation (OR = 0.60, p<.001). Women who were older and those presenting at later gestation were more likely to have serious abortion complications, as were women with induced abortion documented in their medical chart.

**Table 3:** Multiple variable analysis of factors associated with serious abortion complications, adjusted logistic regression odds ratios^a^.

	*Serious complication* *OR (95% CI)*	*Serious complication, among* *induced abortion cases* c *OR* *(95% CI)*	*Sepsis OR (95% CI)*
Time Period			
Before implementation (2001–2003)	Reference	Reference	Reference
Early implementation (2004–2006)	1.0 (0.87, 1.16)	0.85 (0.63, 1.15)	0.60 (0.47, 0.75)***
Later implementation (2007–2010)	0.74 (0.64, 0.85)***	0.49 (0.37, 0.64)***	0.37 (0.29, 0.46)***
Patient age	1.03 (1.02, 1.04)***	1.02 (1.01, 1.04)*	1.05 (1.04, 1.07)***
Induced abortion recorded in chart	6.54 (5.74, 7.46)***	–	15.79 (13.03, 19.13)***
Stage of pregnancy:^b^			
First trimester	Reference	Reference 1.96	Reference
Second trimester	1.78 (1.55, 2.04)***	(1.48, 2.58)***	1.89 (1.52, 2.36)***
Third trimester	1.57 (1.31, 1.88)***	1.77 (1.12, 2.77)*	1.13 (0.81, 1.61)
*Model n*	*21,699*	*1,854*	*21,699*

* p<.05, ** p<.01, ***p<.001.

a All odds ratios are also adjusted for hospital and season.

b Data on the stage of pregnancy was missing in 7.6% of the medical charts. Significance and direction of results were unchanged when the stage of pregnancy variable was excluded from the models.

c Clinical evidence such as foreign body or injury from instrumentation (e.g., uterine perforation) or free-text chart documentation of induced abortion based upon patient disclosure or provider observation.

To confirm results, we also analyzed the trend in serious complications as a proportion of live births at two of the hospitals with available data, and found significant downward trends in the serious abortion complication rate. At MH, there were 6.7 serious complications for every 1000 live births in 2001 which declined relatively consistently to 3.9 serious complications per 1000 live births (Poisson IRR for year = 0.96, p<.001). At LZN live birth data were available starting in 2004, with a serious complications ratio of 11.7 per 1000 live births declining to 1.4 per 1000 live births by early 2011 (Poisson IRR for year = 0.77, p<.001).

## Discussion

Observations in Nepal are instructive for developing countries with high maternal mortality and restricted access to safe abortion care. The declining severity of abortion complications presenting at public referral hospitals, primarily serving poor women most at risk of mortality from unsafe abortion, is evidence that abortion legalization in Nepal has benefited maternal health. Women appear less likely to experience serious complications, and are receiving treatment for complications, likely contributing to reductions in maternal mortality [8], [9], [24]. The overall decline is significant in the most recent phase of implementation, beginning approximately 4 years from the initiation of services, although sepsis complications declined earlier. In Nepal, limited health care infrastructure, challenging terrain, and political instability presented significant challenges for abortion care implementation. The Maoist insurgency in Nepal was at its strongest in 2004, causing major disruptions to roads and supplies just as the safe abortion program began [25]. Nonetheless, persistent expansion of safe abortion care in the face of considerable obstacles appears to have effectively reduced unsafe abortion and its negative health consequences.

The abortion law in Nepal permits abortion on request up to 12 weeks. As a result, women seeking later procedures without a legal indication (*i.e.*, health, fetal anomaly, rape) cannot get care in certified settings and may go elsewhere, limiting the potential of the policy to fully curtail unsafe abortion. Indeed, women seeking abortion at later gestation are at greatest risk of life-threatening complications; we found a greater likelihood of serious complications for cases presenting in the second and third trimester, and the steepest decline in serious complications after initiation of second trimester provider training. Expanding the availability of trained providers, and potentially the limits of the law, may be necessary to further reduce abortion morbidity and mortality in Nepal [26], [27]. Nepal also faces formidable barriers to extending safe abortion care to isolated rural areas, with steep mountain terrain and slow transportation. It is important to support efforts to improve access in these areas, including the training and licensing of lower level health workers to administer medication abortion.

There are several limitations and threats to validity in a hospital-based, natural experiment of this type. Co-occurring demographic, social, and policy changes must be taken into account when interpreting the trend in serious abortion complications at hospitals over a 10-year time period. Use of health care increased over the study period and free maternal health services were instituted in the later years of the study. The overall increase in cases of abortion complications seen at the hospitals could be attributed to underlying trends, such as more abortions obtained after legalization, in both safe and unsafe settings, and/or reduced fear of seeking care for complications. By focusing on high severity cases relative to all cases presenting, we account for broader trends in health care use. Nonetheless, if the likelihood of seeking care for less serious complications increased without a similar rise in seeking treatment for more serious cases, the drop in case severity could be overestimated. Conversely, women with more serious complications that could lead to discovery of induced abortion (i.e., injury) may have been less likely to seek hospital care prior to legalization compared to women with less serious symptoms (i.e., similar to miscarriage). If so, our results would underestimate the decline in serious complications. There is no evidence available to assess whether trends in seeking care differed by severity over the study time period; however, knowledge of the legal status of abortion has remained relatively low, making differential treatment seeking over time less likely [7].

The underlying abortion rate (both spontaneous and induced) is also affected by the fertility rate, contraceptive prevalence, and age distribution of the population. Data from the Nepal Demographic and Health Survey show that from 2001 to 2011, the total fertility rate changed from 4.1 to 2.6 and desired fertility declined. Modern contraceptive prevalence only increased from 35% to 43%, rising until 2006 and since then remaining relatively stable. The proportion of the population of reproductive age also rose. This combination of factors would correspond to more women obtaining induced abortion [28]–[30]. Therefore, the observed decline in case severity seen at hospitals, in the context of more abortions taking place and complications being treated, also could mask a greater decline in abortion morbidity that has occurred in Nepal.

Abortion documentation practices and data quality problems in low-resource settings pose challenges for hospital-based research on abortion [31]. Changes in medical chart documentation practices over the study period would also influence determination of case severity. More detailed descriptions of sepsis and perforation injuries from abortion might be recorded after legalization when patients were no longer subject to prosecution. Evidence from our qualitative study of providers suggests that there may be more detail provided following legalization, and that some code terms for induced abortion are no longer used (we captured these code terms in our abstraction protocol), but that women remain reluctant to disclose abortion even after legalization due to its stigma [19]. Increasing completeness or quality of record-keeping would generally lead us to accurately identify more of the severe abortion complications after legalization, but this would introduce a conservative bias to our trend analysis.

Immediately after abortion became available in Nepal, some types of complications increased, such as uterine injury and shock. The first training center for safe abortion was located at one of the study sites; some of these complications likely arose from newly trained providers, and were promptly treated in the hospital. Additional research and strategy development is needed to help other countries with limited infrastructure address the challenge of quickly training providers and certifying clinics, as rising demand for care in the absence of sufficient numbers of safe providers is likely to occur. A rise in health care use for abortion complications is also to be expected when transitioning to a less punitive policy environment, as fear and stigma related to seeking abortion and health care for complications could decline. At the same time, knowledge about where to obtain safe abortion can be slow to disseminate given communication challenges in settings with low female literacy [32], [33]. Thus, it is important to ensure the availability of sufficient post-abortion care resources immediately post-legalization. The broader context of rising abortion complications at hospitals is also important to consider–when more women receive treatment, maternal mortality may decline. A slight rise in hospital-treated complications may therefore be a positive sign immediately after legalization.

The introduction of medication abortion in Nepal in 2009, and evidence that it has become increasingly available from both certified and uncertified providers may contribute to greater numbers of women presenting with lower severity abortion complications in recent years, as seen in other settings [1], [2]. This shift in mode of abortion likely contributes to the declining abortion complication case severity. Further research into the rising availability of medication abortion and its influence on unsafe abortion and maternal mortality in Nepal could provide important evidence of its role in expanding safe abortion access.

Our results can inform policies on abortion, maternal health and service delivery in low-resource settings where abortion is not legal or where it remains unsafe or inaccessible despite being legal [34]. Legislators and advocates in developing countries with high maternal mortality now have evidence for the public health benefits of policies that support safe abortion care. The model for implementation of safe abortion care in Nepal, involving a coordinated effort by the government, non-government organizations, and private entities, is also validated by our data; other countries may benefit from adapting the approach. The potential need for increased resources for post-abortion care during early phases of implementation, and the importance of second-trimester training is also reflected in our findings. Finally, the legalization of abortion in Nepal was encouraged by public health claims connecting unsafe abortion to high maternal mortality. Results from this study strengthen the link between abortion legalization and improvements in maternal health, as legalization led to reductions in the serious complications from unsafe abortion that contribute to maternal mortality [35]. Legalizing abortion and instituting safe services could help to reduce maternal morbidity and mortality in other settings, and is an important component of efforts toward achieving Millennium Development Goals for countries with high maternal mortality.
